# Data Filtering Method for Intelligent Vehicle Shared Autonomy Based on a Dynamic Time Warping Algorithm

**DOI:** 10.3390/s22239436

**Published:** 2022-12-02

**Authors:** Zhenhai Gao, Tong Yu, Tianjun Sun, Haoyuan Zhao

**Affiliations:** 1 State Key Laboratory of Automotive Simulation and Control, Jilin University, Changchun 130022, China; 2 College of Automotive Engineering, Jilin University, Changchun 130022, China

**Keywords:** autonomous vehicle, discrepancy trigger control, data mining, intelligent vehicle shared autonomy

## Abstract

Big data already covers intelligent vehicles and is driving the autonomous driving industry’s transformation. However, the large amounts of driving data generated will result in complex issues and a huge workload for the test and verification processes of an autonomous driving system. Only effective and precise data extraction and recording aimed at the challenges of low efficiency, poor quality, and a long-time limit for traditional data acquisition can substantially reduce the algorithm development cycle. Based on the premise of driver-dominated vehicle movement, the virtual decision-making of autonomous driving systems under the accompanying state was considered as a reference. Based on a dynamic time warping algorithm and forming a data filtering approach under a dynamic time window, an automatic trigger recording control model for human-vehicle difference feature data was suggested. In this method, the data dimension was minimized, and the efficiency of the data mining was improved. The experimental findings showed that the suggested model decreased recorded invalid data by 75.35% on average and saved about 2.65 TB of data storage space per hour. Compared with industrial-grade methods, it saves an average of 307 GB of storage space per hour.

## 1. Introduction

The development of intelligent driving has entered the era of big data, as shared autonomy has become a crucial step toward autonomous driving. In order to obtain precise information about the traffic environment and vehicle motion states, intelligent vehicles are equipped with various sensors. However, this creates big data disasters, primarily manifested in the data types and dimensions. It is challenging to extract and mine valuable data that discrepancies exist between the driver and the driver assistance system from such a complex mass of data. If the system’s decisions dominate, the driver’s driving experience will be significantly reduced, and traffic accidents may occur. In addition, it is not conducive to the optimization and iteration of the algorithm because it is difficult to collect driving data from drivers under the same operating conditions for a demonstration.

Tesla released the Shadow-Mode technology embedded in its Autopilot system in 2019. The primary function is that, with the Shadow-Mode cloud automatically recording the data on the driver’s daily driving, the autonomous driving algorithm operates in the background with the driver during routine driving and makes real-time decisions but does not control the vehicle. The technology replaces the original test plan of collecting only a few specific experimental cars by sharing the data of all Tesla owners, efficiently enhancing the drawbacks of autonomous driving test scenarios, and offering data support for the next-generation algorithm’s iterative upgrading. The major technical barrier for autonomous driving systems is testing and verification in infinite scenarios. Most China research institutions still use the traditional method of recording experimental data with manual operations, where, to break this significant technological barrier, the consumption of several human resources and time costs while recording redundant and low-quality data with noise is necessary to develop a new generation of autonomous driving systems based on big data on driving behavior and forming a technological chain of iterative upgrading employing data-driven algorithms.

Consequently, a growing number of research institutions and manufacturers have built datasets to accelerate the development of their autonomous driving technologies, such as Honda [1], Audi [2], Baidu [3], Waymo [4,5], the University of Toronto in Canada [6], and the University of California at Berkeley [7]. This is further evidence of the significance of data acquisition technology to the development of autonomous driving.

Autonomous driving data acquisition methods originated from the early scene and driving behavior data collection methods. Scene data were mostly collected using a single camera (e.g., a dashboard camera), and video streaming data from the dashboard cameras were collected for urban 3D scene reconstructions [8,9]. Image data from onboard vehicle cameras were fused with map data to create scene data [10]. The acquisition of driving behavior, the driver’s manipulation of the vehicle, has generally required the addition of specialized sensor equipment. For example, Australian Naturalistic Driving Research [11] and the European Naturalistic Driving Study UDRIVE [12,13] installed numerous customized sensing devices to capture information, including throttle and brake pedal positions and steering wheel angles. Similarly, more sensor devices have been employed for the collection of driving scene data, such as combinations of Lidar, multiple types of cameras, and global navigation satellite systems. Now, autonomous driving data acquisition technology is a fusion of these two types of technologies. Most of the driver’s control command information is read directly through a controller area network (CAN), and wire-controlled chassis technology for the collection of driving behavior data is being continuously developed. For instance, Honda has recorded and combined the data received from global positioning system (GPS), front cameras, accelerometers, CAN buses, and other devices to form the 104-hour Honda Research Institute Driving Dataset [14]. Tesla loaded its shadow-mode technology into a large number of its production vehicles to collect data on driving scenarios and driving behavior that it employs to train its deep learning algorithms [15]. The KITTI dataset integrates data from color and stereo cameras, GPS and inertial measurement unit (IMU) inertial navigation systems, and Lidar to capture a wide range of dynamic and static scenes, both rural and urban [16]. However, most datasets and data acquisition approaches record normal working conditions in conventional scenarios, which means they contain similar data and inadequate characteristics. Moreover, it is the data that has differences between drivers and driver assistance systems that is difficult for algorithms to process and needs to be learned yet locating and capturing this type of data from the vast amount of data available from existing datasets is extremely challenging.

After first-hand collection, the data then have to be processed before being used for autonomous driving algorithm research and development. Common autonomous driving datasets are labeled with information, including obstacles (e.g., pedestrians, bicycles, large commercial vehicles, and small passengers), traffic signs, and traffic lights, to improve the construction, optimization, and validation of perception and decision algorithms [17,18,19]. Since the accuracy of the labels needs to be guaranteed, manual supervision and confirmation are crucial, even with the assistance of artificial intelligence. However, the high volume of raw data leads to high labor and time costs.

**Figure 1:**
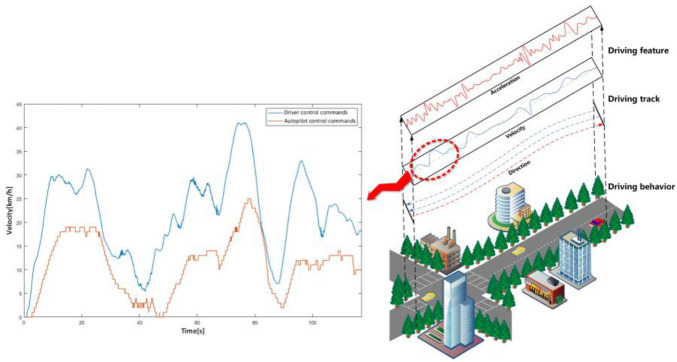
Control command discrepancies between the pilot and the autopilot.

In summary, an analysis of commonly used industry autonomous driving datasets demonstrates that the collected data are highly repetitive with limited effectiveness. These repetitive data were efficient for the initial development of algorithms; however, now, they do not significantly improve the performance of algorithms. The majority of autonomous driving algorithms have already entered optimization and iteration, whereby they can already appropriately handle the majority of repetitive data collected from regular scenarios and normal working conditions. Furthermore, large amounts of redundant data substantially increase the cost of subsequent efforts, such as storage and labeling. Therefore, the primary challenge for autonomous driving data acquisition technology is to effectively and precisely acquire highly targeted and characteristic data to enhance the quality of collected first-hand data and decrease the cost of subsequent work. Whether a pilot or an autopilot drive, the purpose of their driving is the same; however, the pilot and autopilot control commands may differ in the same scenario with the same driving track, as depicted in Figure 1.

By focusing on these challenges, this study presents a discrepancy trigger model based on dynamic time warping (DTW) for the automatic trigger recording of efficient data. Specifically, the contributions of this paper are summarized as follows:

1. We define what highly targeted and effective data from a large amount of driving data is. By analyzing the development process of rule-based and data-driven algorithms, we discover that data with discrepancies between the driving intentions of the driver and the autonomous driving system are effective data after the algorithm enters optimization iteration.
2. We propose a DTW-based discrepancy trigger model to quantify the discrepancy between driving intentions.
3. We design a discrepancy trigger threshold based on statistical principles and combine it with the discrepancy trigger model to achieve an automatic recording of discrepancy data.
4. We validate the effectiveness of the proposed model through simulation and real-vehicle experiments. The experimental results show that the model can reduce the recording of ineffective data by 75.35% on average. Compared to common instantaneous value triggering, it saves an average of 307 GB of data storage space per hour.

This study is organized as follows. In Section 2, the autonomous driving algorithms’ development process is introduced, and the effective data for algorithm optimization is defined. In Section 3, the DTW-based discrepancy trigger model and the computation of the trigger threshold are suggested. In Section 4, simulation and real-world experimental findings are discussed. In Section 5, the primary conclusions and recommendations for future investigation are presented.

## 2. Data Used for Autonomous Driving Algorithm Development

The aim of studying autonomous driving data acquisition approaches is to produce data sets that precisely match the current demands of autonomous driving algorithm development while allowing algorithm developers to rapidly and readily acquire focused data, accelerate the development of autonomous driving algorithms, and reduce any unnecessary duplication of effort. In order to attain these objectives, the processes and demands of current autonomous driving algorithm development are examined in this section.

### 2.1. Autonomous Driving Algorithm Development

The current autonomous driving system solutions include both distributed and centralized system architecture. The distributed system architecture implements autonomous driving functions using four modules: perception, localization, planning, and control. The centralized system architecture implements the autonomous driving function directly using a centralized computing unit [20]. With the recent continuous development of deep learning, research on the latter has gradually increased, and the most typical is end-to-end learning for autonomous vehicles.

In hierarchical system architectures, algorithms frequently employed in the perception module include clustering, support vector machine, and convolutional neural network. Algorithms frequently employed in the localization module include the Particle, Kalman, and Bayesian filters. Algorithms frequently employed in the planning module include search algorithms (e.g., A* and Dijkstra algorithms), random sampling algorithms (e.g., Ant Colony and Round-Trip Time algorithms), and artificial potential field methods. Algorithms frequently employed in the control module include linear quadratic regulator control, model predictive control, and so on. In centralized system architectures, frequently employed algorithms include convolutional neural networks, recurrent neural networks, long short-term memory networks, and combinations of these networks.

**Figure 2:**
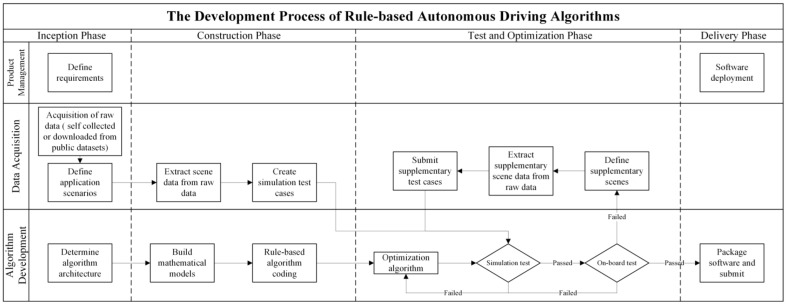
Rule-based algorithm development.

**Figure 3:**
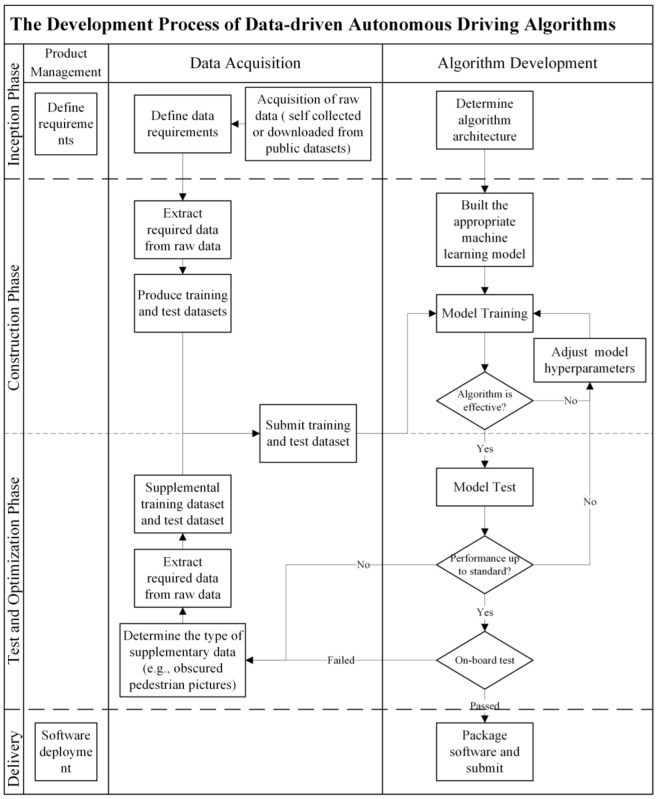
Data-driven algorithm development.

These algorithms were grouped into two approaches: rule-based and data-driven. Figure 2 and Figure 3 show the processes of developing a rule-based autonomous driving algorithm and a data-driven autonomous driving algorithm.

The current autonomous driving algorithm has entered the test and optimization phase. Therefore, the analysis is mainly focused on this phase. The mathematical rules of the rule-based approaches were developed and maximized manually using the algorithm development department. In the test and optimization phases, the algorithm development department first performed simulation experiments on the test cases and maximized the algorithm based on feedback. After the simulation test reached the required standard, an onboard test was conducted. Since the application scenarios of autonomous driving are infinite, algorithms may encounter scenarios that are not included in simulation cases during real-world testing and cannot be solved. In this situation, the algorithm development department abstracted and expressed the scenarios before returning them to the data acquisition department, and the data acquisition department collected the significant scenario data and produced novel simulation cases. After the test cases were supplemented, the above steps were repeated until the real vehicle test was passed.

Data-driven approaches, generally called machine learning approaches, are where mathematical rules are extracted and maximized from the data by the models. In the test and optimization phase, the algorithm development department first conducted the algorithm with the test dataset and further optimized the algorithm’s hyperparameters based on the feedback findings. If the algorithm was consistently unable to solve a certain class of challenges, the algorithm development department collated the class of challenges and sent them back to the data acquisition department. The data collection department then performed a new round of data feature engineerings, such as data gathering and extraction, and returned the supplemented data set to the algorithm development department for further training. The algorithm was cycled in this way until the predicted performance was achieved in both test sets and real vehicle experiments.

In summary, due to the infinite nature of driving scenarios, data acquisition and processing were ongoing throughout the development of autonomous driving algorithms. Efficient data acquisition methods substantially improve algorithm design, and high-quality datasets directly influence the final performance of an algorithm. Therefore, data acquisition and processing have had a crucial influence on the development speed of algorithms, and high-quality raw data will substantially reduce the data processing effort. The next subsection further introduces the high-quality raw data required for current algorithm development in the context of the algorithm development process.

### 2.2. Data Required for Autonomous Driving Algorithm Development

In the early years, SAE International in the USA classified autonomous driving into five levels. As research gradually progressed, predictions for autonomous driving technology grew from achieving simple autonomous driving to attaining comfortable and safe human-like driving to eventually achieving driving that is more comfortable and safer than human driving. Existing self-driving technologies have achieved simple autonomous driving, but their driving ability has not yet reached the level of human driving, as is explained by several driving scenarios that humans can solve but autonomous driving systems cannot. Accordingly, finding various daily driving scenarios that autonomous driving systems cannot solve is a crucial problem because current autonomous driving technologies are no longer in the inception and construction phases; the algorithms are in the testing, optimization, and iterative upgrading phases. Therefore, effective data for iterative optimization is scenario data that autonomous driving systems cannot deal with and the correct solutions to those scenarios (e.g., proper control commands and correct obstacle categories).

The following is an approach to distinguishing scenarios that cannot be solved by autonomous driving algorithms in the process of collecting routine driving data. Since the driving ability of the current autonomous driving system does not surpass that of humans and the autonomous driving system’s driving style should imitate humans to all possible extents, humans’ normal driving behavior (excluding the behavior that results in accidents) could be considered the standard behavior in current driving scenarios. A driver’s driving behavior is certainly safe or at least cautious in the case of autonomous driving data acquisition, so during the data acquisition process, the driver’s driving intentions and driving behaviors can be directly employed as standard answers for current scenarios. This indicates that the algorithm cannot solve the current scenario, so the algorithm is deemed in error when the decisions made by the algorithm are sufficiently distinct from the driver’s decisions.

**Figure 4:**
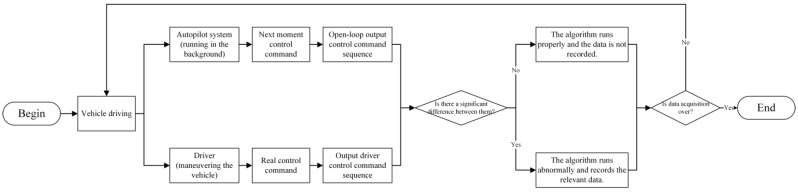
Process of identifying scenarios that are difficult for the autopilot system to handle.

Figure 4 shows the process of identifying scenarios that are too difficult for the autopilot system to handle. It is important to note that the autopilot system plans a sequence of control commands in a time horizon, but we only extract the control commands for the next moment. The reason for this is that only the next moment’s control command is calculated based on the current environment, and subsequent control commands within the period are planned based on predictions for the future. However, such predictions are meaningless in the case where the driver is actually controlling the vehicle. We have no way of knowing the driver’s prediction and planning of the future trajectory, and therefore we cannot compare the subsequent control commands in the time horizon with the intention in the driver’s mind. To circumvent this problem, we only record the next command given based on the current scene, which for the autonomous driving system is the first control command in the time horizon, and for the driver, the command actually executed in the next moment. It makes sense to compare two commands such as this. However, comparing commands from just one moment does not show the difference in trend between control commands over a time horizon. To solve this problem, we synthesize commands from a past time horizon into a sequence to compare and analyze the trend discrepancy. When there is a large discrepancy in the sequence of control commands between the autopilot system and the driver in the past time horizon, it triggers the recording of the data in the period.

**Figure 5:**
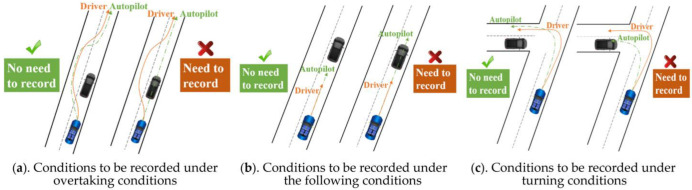
Examples of scenarios where data needs to be recorded.

This paragraph presents a qualitative analysis of the magnitude of the difference in the decisions made by the driver and the autonomous driving system. The purpose of driving is to regulate a vehicle to the desired location according to the desired spatiotemporal trajectory. This means that the driving behavior is significantly a control behavior. Therefore, the algorithm and driver decisions can be characterized using the control instructions they output. When the difference between their control instructions is sufficient to cause the vehicle to deviate substantially from the desired trajectory in space-time, the decisions between the autonomous driving algorithm and the driver are regarded to differ to a large extent. It further shows that the scenario at that moment cannot be suitably solved by the autonomous driving algorithm; therefore, the current and past periods’ relevant data are efficient and need to be recorded, as depicted in Figure 5 Section 3 introduces the discrepancy quantification method between control commands.

## 3. Discrepancy Trigger Model Based on the Dynamic Time Warping Algorithm

This section presents the control command discrepancy trigger model and quantifies the discrepancy between driver and autopilot control commands by computing the distance between two control command sequences over a period of time. Two sequences of commands with the same control findings may also encounter the challenge of misaligned time axes due to the differences in control cycles and driver and autopilot modes. Therefore, the DTW algorithm was introduced to compare two sequences where the time axes are not strictly aligned.

### 3.1. Dynamic Time Warping Algorithm

**Figure 6:**
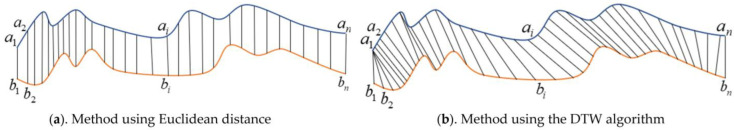
The differences between two methods in distance calculation.

The DTW algorithm, first suggested by Itakura, a Japanese scholar, is used to determine the similarity of two time series of different lengths [21]. Compared to methods that employ Euclidean distance to determine similarity, DTW is a more robust approach that can conduct the similarity matching of curve shapes even when they are not synchronized in time [22,23]. The sequence of control commands outputted by the driver and the autopilot system is challenged by time asynchrony since their control cycles are not similar. Thus, the DTW method was selected to quantify the spatiotemporal similarity of the two control command curves. The two sequences had shape similarities, but their time axes were not aligned. The Euclidean distance forces the first and last points in one sequence to align with the first and last points of another sequence, which leads to similar shapes in both sequences not being correctly matched and a resulting unsuitable measure. However, DTW matches similar shapes more accurately. Figure 6 shows the differences between the two approaches.

The principle of DTW is described below. A and B represent two time series of length n and m, respectively, as defined in Equation (1):

(1) $$\begin{array}{l} A=a_{1},a_{2},\cdots ,a_{i},\cdots ,a_{n}\\ B=b_{1},b_{2},\cdots ,b_{j},\cdots ,b_{m} \end{array}$$

The DTW algorithm first constructs n using m a distance matrix D, where the ($i^{th}$, $j^{th}$) element of the matrix D denotes the distance $d\left(a_{i},b_{j}\right)$ between point $a_{i}$ and point $b_{j}$. The distance is computed in Equation (2):

(2) $$d\left(a_{i},b_{j}\right)={\left(a_{i}-b_{j}\right)}^{2},$$

The warping path W denotes the set of distance matrix D elements, which denote the correspondence of points on series A and series B. The $k^{th}$ point on W is defined as representing the $i^{th}$ point of series A, which matches with the $j^{th}$ point of series B. Figure 5 shows the distance matrix D and the warping path. Equation (3) defines:

(3) $$\begin{array}{l} W=w_{1},w_{2},\cdots ,w_{k},\cdots ,w_{H}\;\begin{array}{c} \max\left(m,n\right)\leq H\leq m+n-1 \end{array}\\ w_{k}=D_{i,j}={\left(a_{i}-b_{j}\right)}^{2} \end{array}$$

The DTW algorithm constructs the distance matrix and searches it for the optimal warping path. Equation (4) defines the cost C of the warping path as follows:

(4) $$C=\sum_{k=1}^{H}w_{k},$$

Equation (5) shows that the solution of the DTW can be considered an optimization problem: to discover the warping path with the minimum cost.

(5) $$DTW\left(A,B\right)=\min C,$$

The warping path generally meets the following three constraints [22]:

1. Boundary constraint: $w_{1}=D_{1,1}$ and $w_{H}=D_{m,n}$. This limitation requires that the warping path starts from the lower left corner of the distance matrix and ends at the upper right corner. This ensures that the two-time series performs similarity matching from beginning to end.
2. Continuity constraint: if $w_{k}=D_{a,b}$, $w_{k-1}=D_{a^{\prime },b^{\prime }}$, then $a-a^{\prime }\leq 1$, and $b-b^{\prime }\leq 1$. This limitation requires that the warping path can only reach adjacent cells (including diagonally adjacent) at each step, ensuring that similarity matching is performed at each point in the two-time series.
3. Monotonicity constraint: if $w_{k}=D_{a,b}$, $w_{k-1}=D_{a^{\prime },b^{\prime }}$, then $a-a^{\prime }\geq 0$, and $b-b^{\prime }\geq 0$. This limitation ensures that the warping path is monotonic in time, which guarantees that one shape in a time series will not be repeatedly matched.

This brief introduction to the DTW algorithm and this study employed MATLAB software to solve the DTW. The following describes the DTW-based control command sequence discrepancy trigger model.

### 3.2. Trigger Model Based on Control Command Discrepancy

**Figure 7:**
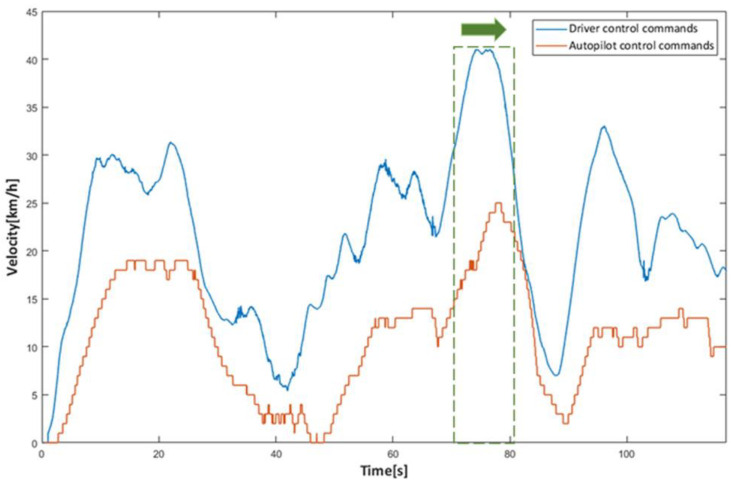
Scroll time window.

In order to thoroughly determine the differences between the driver and autonomous driving system control commands, the suggested discrepancy trigger model was developed with two independent pathways to quantify the differences in longitudinal and lateral control commands. Since the autonomous driving algorithms’ output and types of longitudinal control commands differed, the common measurable acceleration or vehicle speed was selected as the input for the longitudinal control command analysis. The steering wheel angle was chosen as the input for the lateral control command analysis pathway. Figure 7 shows that at a single moment, the model input was not the control command value but a sequence of control commands over some time that scrolled forward in a time window. The length of the time window was the duration of the intercepted sequence of control commands, which could be adjusted according to the specific application scenario. In this study, to completely record the driving action within a driving scenario, the duration of all four input sequences (two-speed sequences and two steering wheel angle sequences for the driver and the autopilot system) was 10 s.

The distance matrices D and d of the longitudinal input path and the lateral input path were computed according to Equation (2). The optimization challenge was solved to discover the warping path with the minimum warping cost, and the warping cost of this path was compared to the preset threshold value. If the warping cost was higher than the threshold value, this demonstrated that the driving scenario could not be properly handled using the automated driving system, and the relevant data should be recorded, thus showing that there was a large difference in the control command sequence between the driver and the automated driving system.

The following describes the selection and calculation of the threshold value. The elements in the distance matrix D, which are the distances between the points on the two input sequences, can be considered independent and identically distributed random variables and expressed as $X_{i}$. According to the central limit theorem, it can be concluded that $\sum X_{i}$ approximately obeys a normal distribution. The warping cost $C=\sum X_{i}$, so it can be concluded that C approximately obeys normal distribution, as depicted in Equation (6):

(6) $$C=\sum X_{i}∼N(\mu ,\sigma ),$$

According to a normal distribution, 68.26% of the data were in the interval $\left(u-\sigma ,u+\sigma \right)$. The probability of the occurrence of warping costs greater than u+σ is statistically less than or equal to 15.87%. In this study, it was assumed that scenarios not handled properly or not able to be handled by the autonomous driving system accounted for about 15% of the total scenarios based on experience and the current state of the industry. Therefore, the threshold was calculated as follows in Equation (7):

(7) $$t_{H}=\frac{\sum_{i=1}^{n}C_{i}}{n}+\sqrt{\frac{{\sum_{i=1}^{n}\left(C_{i}-\frac{\sum_{i=1}^{n}C_{i}}{n}\right)}^{2}}{n}},$$

where $C^{i}$ denotes the warping cost of the $i^{th}$ test and n represents the number of tests. It should be noted that the thresholds applied to the longitudinal pathway and the lateral pathway were not the same. They were selected and calculated independently since the longitudinal and lateral motion control of a vehicle are very different.

**Figure 8:**
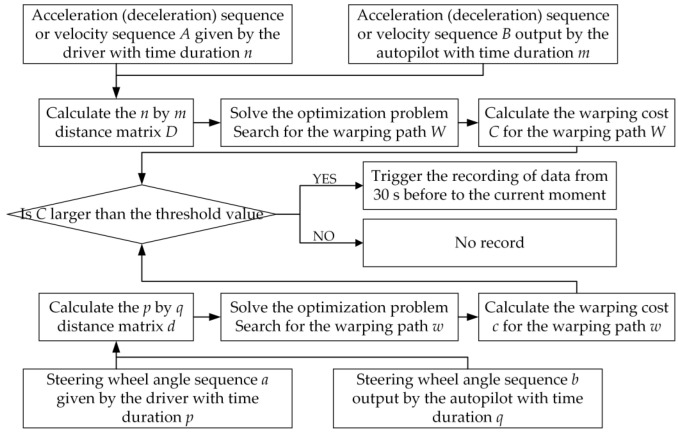
Flowchart of the DTW-based control command discrepancy trigger model.

Finally, a method for data recording was introduced. All the data types that needed to be recorded after a trigger were refreshed with a time window sliding forward. If data recording was not triggered in the current time unit, the time window slid forward one unit, and the data at the latest moment was incorporated into the time window for temporary storage while the data at the time window’s end was cleared out. This approach ensured that the driving actions and scenes were recorded in their entirety while decreasing the minimum storage space requirements for the device. This time window data recording approach can be readily implemented on a computer using a queue storage structure and enqueue and dequeue operations. A flowchart of the DTW-based control command discrepancy trigger model is shown in Figure 8.

## 4. Experiments and Results

**Figure 9:**
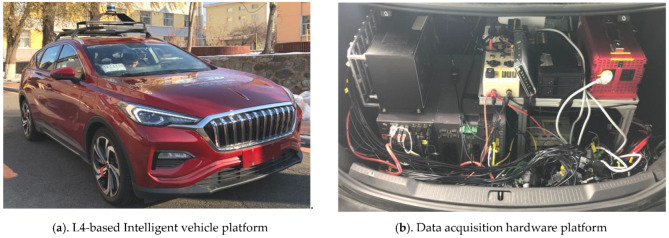
The real vehicle test platform.

In this study, simulation and real-world tests were performed on the basis of a passenger car equipped with a Cincoze DS1201 which was equipped with ROS Kinetic and MATLAB R2018b (as shown in Figure 9). The driver controlled the vehicle, and the autonomous driving algorithm was placed in the background and operated during the test. In the simulation test, data from the driver and the autonomous driving algorithm were recorded, and the discrepancy trigger model conducted an offline discrepancy trigger of the recorded data in a separate device. In the real-world test, to conduct online discrepancy trigger data recording, the discrepancy trigger model operated in real-time in the onboard IPC. In this study, the common L2-level adaptive cruise control (ACC) algorithm and the L4-level autonomous driving algorithm on a closed campus were chosen as the tested algorithms.

In order to confirm the discrepancy trigger model’s effectiveness, the test scenarios intentionally increased uncertainty to ensure adequate discrepancies between the driver and the autonomous driving algorithm. For the ACC algorithm, a free-following scenario was developed for the testing, in which the driver randomly switched driving styles when following a car. For the L4 autonomous driving algorithm, a tracking scenario on the campus was developed, with the tracking route passing through intersections and areas with numerous pedestrians.

### 4.1. Simulation Tests

In order to compare the trigger mechanism based on the discrepancy trigger model; a trigger mechanism based on instantaneous discrepancy values was chosen. A control command sequence was plotted versus time to visually depict the difference between the various triggering mechanisms in terms of discrepancy detection and data logging. The solid line represents the portion where the model identified a discrepancy, and there was trigger data logging, while the dashed line represents the portion where the model identified no discrepancy, and there was no trigger data logging, with the two separated by a vertical green dashed line. The curves provided a clear and complete picture of the driving actions of the driver and the autonomous driving system; therefore, the accuracy of the discrepancies detected by the models could be readily determined. The longitudinal control command discrepancy trigger test was performed based on the ACC algorithm, and the vehicle velocity was selected as model input because the control command output of the equipped ACC algorithm was the vehicle velocity. The lateral control command discrepancy trigger test was performed on the basis of the L4 autonomous driving algorithm, and the steering wheel angle was selected as the model input.

**Figure 10:**
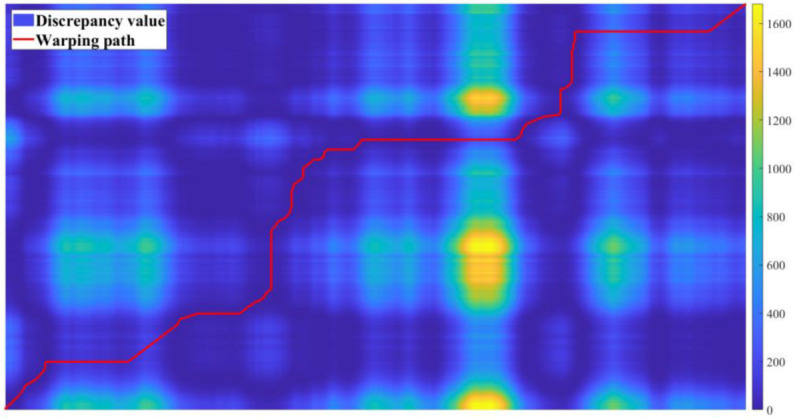
Distance matrix and warping path visualization for the 1st round test.

**Figure 11:**
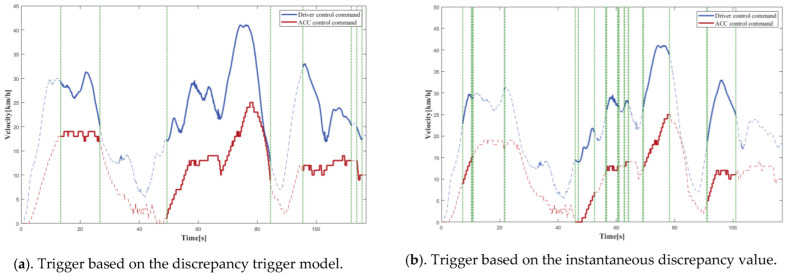
The 1st round test results for longitudinal control command discrepancy data trigger recording.

**Figure 12:**
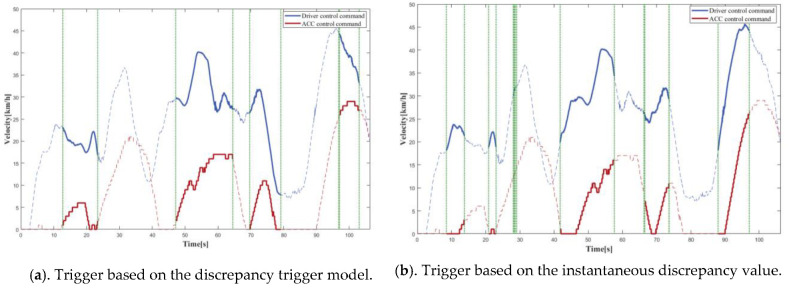
The 2nd round test results for longitudinal control command discrepancy data trigger recording.

**Figure 13:**
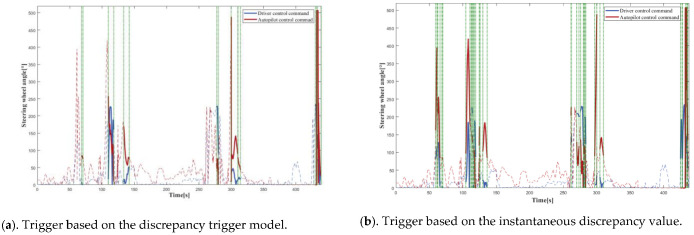
The 1st round test results for lateral control command discrepancy data trigger recording.

**Figure 14:**
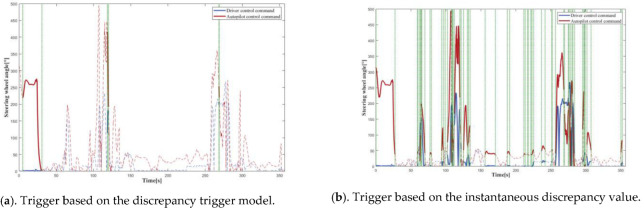
The 2nd round test results for lateral control command discrepancy data trigger recording.

Figure 10 depicts the distance matrix D and the warping path W computed by the proposed model for the first round of longitudinal control command data triggering tests. Figure 11, Figure 12, Figure 13 and Figure 14 present the discrepancy trigger findings of the two discrepancy trigger approaches for longitudinal and lateral control commands, which were assessed independently over two rounds. The findings indicated that the proposed model could detect substantial discrepancies between the driving intentions of the driver and the autonomous driving system and record the relevant data segments completely, which efficiently enhanced the quality of the recorded data and reduced the total data collection amount. Compared to the instantaneous value-based discrepancy trigger method, the DTW-based discrepancy trigger model could sensitively capture and completely record situations where the control commands of the driver and the autonomous driving algorithm had distinct or even opposite trends in the longitudinal direction. Its triggering was also more precise in the lateral direction.

### 4.2. Real Vehicle Tests

In order to confirm the proposed model’s effectiveness in the real world, it was configured in the vehicle IPC, and Simulink software was employed to communicate with the ROS system to attain real-time discrepancy detection and data-trigger recording in the real vehicle. According to the experimental findings, the control commands output of the driver and the ACC algorithm were plotted versus time, where the dashed line is all the data during the test, and the solid line is the data recorded by the system after applying the discrepancy trigger model, using a vertical green dashed line to distinguish the two.

**Figure 15:**
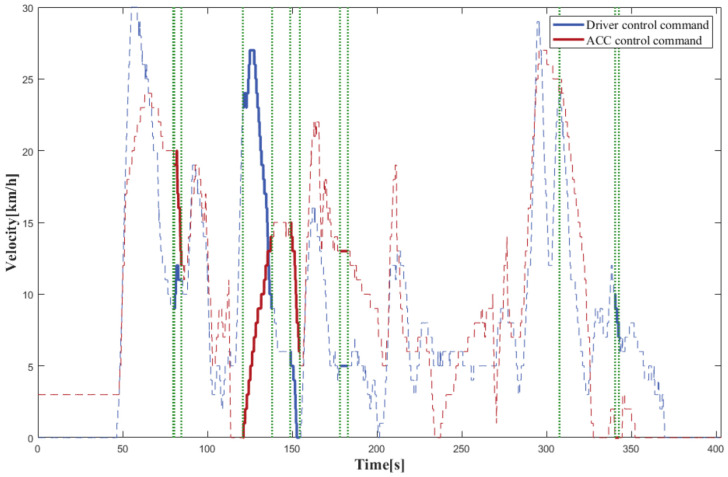
The results for real vehicle test.

As depicted in Figure 15, the findings demonstrated that the suggested model was equally efficient in the real world. This satisfied the real-time requirements for online operating in real-time for the onboard IPC, and the discrepancy trigger model, when operating on the real vehicle, was also capable of precisely detecting the existence of different or opposite trends in the control commands of the driver and the autopilot algorithm and recording the significant data completely.

To quantitatively examine the model’s performance, this study computed the reduction in recorded data following the application of the discrepancy trigger model. Since the amount of data recorded per hour differed among various autonomous driving systems, the data recording reduction rate δ was computed using the data recording time as a metric, as shown in Equation (8) as follows:

(8) $$\delta =1-\frac{t_{r}}{t},$$

where t denotes the total test duration and $t_{r}$ represents the triggered recording duration. Before applying the model, all test times were recorded after applying the model. However, only the triggered periods were recorded; therefore, δ could characterize the percentage reduction in data recording after employing the discrepancy trigger model.

Table 1 presents the findings of the quantitative analysis of the multiple tests. The findings show that the amount of data recording was reduced by 75.35% on average after applying the discrepancy trigger model. Based on the projection that the autonomous driving system produces 3600 GB of data per hour, the discrepancy trigger model can save 2.65 TB of data storage space per hour. Compared with the instantaneous value triggering, the model reduces the recording of data by 8.61% on average, saving 307 GB of storage space per hour.

## 5. Conclusions

To solve the problems of high data dimensionality and data redundancy in the iterative process of intelligent vehicle system optimization, we designed an automatically triggered data filtering method based on a DTW algorithm after considering the lack of discrepancy identification in traditional data collection and storage approaches. We discovered that there were inconsistencies between the driver’s control behavior and the output of the behavioral decisions by the driver assistance system or the autonomous driving system for the same traffic scenario. These discrepancies could be quantitatively distinguished by quantification. We then constructed an auto-triggered record control model based on a dynamic time warping algorithm to circumvent data with a low human-vehicle discrepancy in the case of shared autonomy. Finally, the effectiveness of the model was confirmed using simulation and real-vehicle tests. In disparate, characteristic, high-quality data offering theoretical support for subsequent iterations of the autonomous driving system, the quantitative analysis demonstrated that the suggested model records an average of 75.35% less data, which will save 2.65 TB of data storage space per hour. Compared to the frequently used instantaneous difference value trigger, it saves an average of 307 GB of storage space per hour.

The longitudinal and lateral control commands were in two separate paths, meaning that their discrepancies were computed separately, and the corresponding data logging was triggered independently. However, during actual driving, longitudinal and lateral control commands are often somewhat coupled, so independently computed discrepancies are sometimes inaccurate. We plan to examine the discrepancy detection model with lateral and longitudinal fusion to further enhance the model’s accuracy in detecting differences in driving intentions and to increase the quality and effectiveness of the data collection.

## Figures and Tables

**Table 1:** Quantitative analysis findings.

Test Rounds	Total Test Duration (s)	Triggered Recording Duration (s)	Data Recording Reduction Rate (%)	Projected Storage Space Savings per Hour (TB)
First test	120	66.64	44.47	1.56
Second test	106	43.60	58.57	2.06
Third test	439	43.92	90.00	3.16
Forth test	357	27.76	92.22	3.24
Fifth test	404	34.40	91.49	3.22
Average value			75.35	2.65

## Data Availability

The data presented in this study are available on request from the corresponding author. The data are not publicly available due to privacy.
